# A Morphometric Analysis of Platelet Dense Granules of Patients with Unexplained Bleeding: A New Entity of Delta-Microgranular Storage Pool Deficiency

**DOI:** 10.3390/jcm9061734

**Published:** 2020-06-04

**Authors:** William T. Gunning, Meera Raghavan, Edward P. Calomeni, James N. Turner, Bodri Roysam, Shantala Roysam, Mary R. Smith, Peter A. Kouides, Neil A. Lachant

**Affiliations:** 1 Department of Pathology, University of Ohio, Toledo, OH 43614, USA; edward.calomeni@osumc.edu; 2 Kansas City University of Medicine and Biosciences, Kansas City, MO 64106, USA; mraghavan@kcumb.edu; 3 New York State Department of Health, Albany, NY 12203, USA; jturner@binghamton.edu; 4 Department of Electrical and Computer Engineering, University of Houston, Houston, TX 77204, USA; broysam@central.uh.edu; 5 Independent Researcher, Houston, TX 77002, USA; shantala.roysam@gmail.com; 6 Department of Medicine, University of Toledo, Toledo, OH 43614, USA; maryrsmith@utoledo.edu; 7 Mary Gooley Hemophilia Center, Rochester General Hospital, Rochester, NY 14621, USA; peter.kouides@rochesterregional.org; 8 Wilmot Cancer Institute, University of Rochester Medical Center, Rochester, NY 14642, USA; Neil_Lachant@URMC.Rochester.edu

**Keywords:** unexplained bleeding, platelet storage pool deficiency, platelet dense granules

## Abstract

One thousand and eighty patients, having prolonged bleeding times, frequent epistaxis, menorrhagia or easy bruising or other bleeding manifestations, and excluding those with von Willebrand’s disease, were evaluated for platelet dense granule deficiency. The mean diameter of platelet dense granules was determined for all patients using image analysis. Four hundred and ninety-nine had “classic” dense (delta) granule storage pool deficiency (δ-SPD). Five hundred and eighty-one individuals (53.8%) were found to have a normal mean number of dense granules, but for some of these patients, the dense granules were smaller than for the controls. Of the patients having a normal number of dense granules, 165 (28.4%) were found to have significantly smaller granules than the platelets obtained from the control subjects. Their average granule diameter was 123.35 ± 0.86 nm, that is more than three standard deviations below the mean of the control data. Total δ-granule storage pool volumes (TDGV)/platelet were calculated using these measurements. Individuals with δ-SPD had half the number of granules (2.25 ± 0.04 DG/PL) and storage pool volume (3.88 ± 1.06 × 10^6^ nm^3^) when compared to our control data (4.64 ± 0.11 DG/PL; 10.79 × 10^6^ nm^3^ ± 0.42). Individuals having a bleeding history but a normal average of small dense granules had a calculated storage pool volume statistically different than controls and essentially the same storage pool volume as patients with δ-SPD. We have identified a sub-classification of δ-SPD that we have defined as micro-granular storage pool deficiency (δ-MGSPD).

## 1. Introduction

Storage pool deficiency (SPD) is a heterogeneous bleeding disorder related to a decreased number of specific platelet organelles resulting in platelet dysfunction. Deficiencies of platelet alpha granules (α-SPD), dense (delta) granules (δ-SPD) and combined deficiencies of both alpha and delta granules, including their respective substance contents, will all manifest with common bleeding symptoms. The clinical presentation of a patient with SPD often includes a history of easy bruising, frequent epistaxis, surgical bleeding, heavy menses and/or post-partum hemorrhage with a normal or prolonged screening test of platelet function in terms of the bleeding time or closure time with or without abnormal aggregation tests. Both hereditary [1,2,3,4,5,6,7,8] and acquired [9,10] etiologies have been reported. Chédiak–Higashi [1,11], Hermansky–Pudlak [12,13,14,15], and Wiskott–Aldrich [16] syndromes are examples of hereditary δ-SPDs. Acquired δ-SPDs have been reported in subjects from myeloproliferative disorders [17] cirrhosis [18,19], and uremia [20,21,22].

Patients presenting with a history of bleeding predominantly affecting the skin, mucous membranes, gastrointestinal, gynecological, and urinary tracts, or who have had a significant post-surgical bleeding complication, should be evaluated for a platelet quantitative or qualitative disorder. It is not uncommon for patients to remain without a diagnosis following platelet aggregation studies if von Willebrand antigen and activity levels and factor VIII clotting activity are all normal. In such instances, δ-SPD should be considered a potential etiology. Nieuwenhuis et al. reported that of 106 patients diagnosed with δ-SPD, 23 individuals (13 congenital and 10 acquired δ-SPD) had normal aggregation responses to ADP, epinephrine, and collagen [23]. A similar study by Israels et al. reported that of 153 new patients referred for a work-up of abnormal bleeding had a prolonged bleeding time and of these, 46 were evaluated for platelet dense granule deficiency and decreased ATP release [24]. Ten percent (5/46) were found to have a dense granule deficiency, yet had no aggregation defect [24]. Both groups of investigators, using a normal reference range of 3–8 dense granules per platelet (DG/PL), concluded that δ-SPD is common, heterogeneous and cannot be excluded using aggregation assays alone. We have similar unpublished data that was presented at an International Society on Thrombosis and Haemostasis meeting in Toronto, Canada [25] and has been confirmed in a report by Brunet et al. [26]. More recently, others have reported that dense granule reference ranges may vary by age group [27].

We include the electron microscope (EM) as an essential tool to evaluate patients with unexplained bleeding for potential dense granule deficiency (δ-SPD), using the platelet whole mount technique originally described by Bull [28] and refined by White [12,13,24,29]. Our methodology follows classic protocols that have been established in the literature over the past five decades; we do not have energy dispersive X-ray (EDX) available to use when evaluating platelets. Initially, we identified a cohort of patients (*n* = 13) with bleeding and elevated numbers of dense granules (δ-SPD); nine of whom had an abnormal aggregation response to either ADP or epinephrine. Many of these patients had significantly smaller DGs than those of normal subjects. Subsequently, we tested the hypothesis that the determination of the DG size and volume may be useful in the evaluation of patients with significant bleeding symptoms with normal to elevated numbers of DGs and who were not found to have von Willebrand disease (VWD). We report a variant of δ-SPD that we have termed microgranular (delta) storage pool deficiency (δ-MGSPD).

## 2. Materials and Methods

### 2.1. Index Patients

This study was based upon the clinical and laboratory observations of 13 individuals seen at our hospital who presented with abnormal bleeding and unusual laboratory test results, including elevated numbers of platelet dense granules enumerated by the utilization of platelet whole mount electron microscopy. The patient demographics, including 9 females and 4 males, whose ages ranged from 13 to 80 years, are shown in Table 1. Nine out of 13 had a family history of bleeding. The ethnic make-up included 10 Caucasians (2 Eastern European Jewish), 1 African American, 1 Hispanic and 1 Laotian. The severity of the bleeding ranged from easy bruising and menorrhagia to life threatening bleeding after invasive procedures. While many had been treated with fresh frozen plasma, cryoprecipitate or vitamin K for their bleeding episodes, none had been empirically treated with platelet transfusion or with desmopressin (DDAVP). Six of the 13 individuals had a bleeding time (BT) that was prolonged at >8.5 min (BTs were done with a Simplate device, with the cuts parallel to the antecubital crease). There was no correlation between the bleeding time and the extent of clinical bleeding. Twelve individuals had platelet aggregation studies performed (the 13th specimen clotted and was not reordered). As has been reported for dense granule deficiency [12,23], the aggregation response was variable. All had a normal aggregation response to 1 and 4 µg/mL of equine tendon collagen. Four individuals had a normal response to both ADP and epinephrine, while 4 had a suboptimal response to either ADP or epinephrine, and 5 had a suboptimal response to both ADP and epinephrine. During the assessment by transmission electron microscopy (TEM), their platelet dense granules appeared to be smaller than usual. There was no correlation between the aggregation response to ADP and epinephrine and the dense granules size. We were unable to assess the subsequent cases included in this study for correlations as the majority of cases were submitted from a number of different institutions without accompanying clinical and laboratory test data.

### 2.2. Samples and Standards

This is a descriptive case series that utilized the blood collected from 49 healthy adults, used as quality control donors for aggregation assays, to establish control values of the platelet dense granule number, dense granule diameter and the concentration of adenine nucleotides extracted from platelets to determine the ATP:ADP ratios. We presented the data collected over a twenty-year period from 1080 individuals with unexplained bleeding, suggestive of a platelet function disorder, who had been assessed for diagnostic purposes using whole mount TEM (wmTEM), dense granule diameters, and the infrequent determination of adenine nucleotide ratio. Many subject blood samples originated from institutions other than the authors’ institutions and for most samples, clinical symptoms and associated laboratory assessment results were not provided. All the samples included in this study were submitted to our laboratory for the evaluation of the unexplained bleeding. When the clinical history was provided, all the subjects had reported symptoms suggestive of a platelet function disorder or other hemostasis defect. Platelet dense granule stability in whole blood acid dextrose citrate vacutainers and shipped via air freight to our laboratory was unaltered if received within 48 h of venipuncture [30]. To validate the dense granule stability in the blood samples that were shipped to our laboratory, we compared the mean number of DGs/PL in whole blood samples obtained from 35 healthy student volunteers, all in their 20s with a balance of both sexes. Two vials were drawn, with one sample analyzed immediately after venipuncture and another one shipped airfreight to Kansas City, Missouri. Each shipment (each contained 5–7 samples with 1 shipment per week) was immediately reshipped to our laboratory and processed 48 h post-venipuncture for comparison. There was no significant difference between the dense granule number in the samples assessed at the day of venipuncture (4.4 ± 0.2 DG/PL) compared with blood that was shipped and analyzed 48 h post-venipuncture (4.3 ± 0.2 DG/PL) [30]. The results were also within the normal range of DG/PL as established in the literature [13,25]. All the procedures were performed using Internal Review Board (IRB)-approved protocols as specified by Federal guidelines and standards and conforming to the rules of the Declaration of Helsinki of 1975.

### 2.3. Platelet Rich Plasma (PRP) Preparation for Electron Microscopy of Platelet Whole Mounts

Peripheral blood was acquired from subjects by routine venipuncture and placed into 7 mL or 10 mL ACD (acid citrate dextrose) Vacutainer^®^ brand evacuated blood collection tubes containing 22.0 mg/mL trisodium citrate, 8.0 mg/mL citric acid and 24.5 mg/mL dextrose. Platelet rich plasma (PRP) was obtained by centrifugation at 100 g for 15 min. Each vial of centrifuged blood was incubated at 37° in a sand bath for 30 min, allowing a “rest” period for the platelets prior to the subsequent processes. The determination of the PRP platelet count was made using a Coulter Hematology Analyzer (STK-R or S, Beckman Coulter, Inc., Fullerton, CA). Aliquots of each sample were processed for whole mount transmission electron microscopy (wmTEM) and the extraction of adenosine nucleotides. These samples were not utilized for light transmission aggregometry (LTA).

### 2.4. Whole Mount Technique

**Figure 1:**
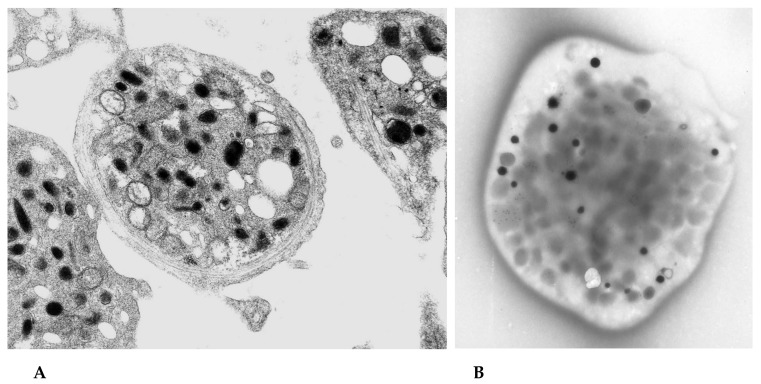
Normal platelets obtained from the peripheral blood. (**A**) Thin section of the epoxy-embedded platelets demonstrates a number of various organelles. Dense granules are not readily seen in the sections of platelets as not all will be within the plane of the section; the majority of the “dark” bodies are alpha granules. (**B**) A whole mounted and air-dried platelet includes all the granules within the cell. Dense granules appear opaque black due to the calcium content of these organelles that vary in both size and density. Many obscure gray shadows may be seen; these represent the alpha granules that are not electron dense and are in the number range of 50–80 per platelet.

The whole mount technique for the enumeration of platelet delta granules has been previously described [13,28,31,32]. In summary, drops of PRP were placed upon multiple electron microscope (EM) grids coated with parlodion and stabilized with evaporated carbon, for 5–10 min at room temperature (approximately 20 µL for each grid). The platelets adhered to the surface of the carbon-coated plastic support film without becoming activated. Subsequently, the grids were blotted with bibulous paper to remove the excess PRP and were “washed” by placing the grids upon drops of distilled water briefly and blotted again; the wash step was repeated three times. Platelet whole mount preparations were allowed to air dry before proceeding to the TEM. Dense granules were readily identified in air-dried whole cell preparations due to their calcium content in contrast to the epoxy-embedded and sectioned platelets that are representative of only a thin sliver of a platelet’s cytosol with other organelles, including alpha granules and lysosomes, also having electron density imparted via heavy metal fixation techniques and post-section staining (Figure 1).

### 2.5. Routine TEM, High-Voltage Electron Microscopy, and Uranaffin Reactions

Once the aliquots of PRP were acquired for the preparation of whole mounts and extractions, an additional centrifugation at 500× *g* for 15 min and the removal of the plasma layer were conducted, the overlaying buffy coat was fixed with 3% glutaraldehyde fixative and buffered with 0.2 M sodium cacodylate for 2 h. Subsequently, the fixed buffy coat was divided and processed for the routine embedment for transmission electron microscopy (TEM), high-voltage transmission electron microscopy (HVEM) and an uranaffin reaction [12].

**Figure 2:**
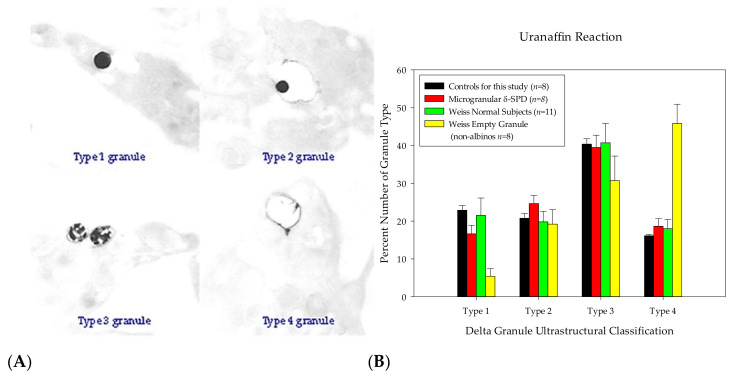
The uranaffin reaction demonstrates the four stages of dense granule development. (**A**) Epoxy-embedded platelets that reacted with uranyl acetate were categorized into 4 stages of development with Type 1 granules being fully developed; an empty vacuole (Type 4 granules) represents the beginning of the earliest phase of a dense granule. (**B**) This graph demonstrates the distribution of dense granule types for the control (normal subjects) and microgranular storage pool deficiency presented in this report. The data for our control and microgranular (delta) granule storage pool deficiency (δ-MGSPD) subjects are identical to the 4 stages of dense granule development in normal patients as described by Weiss and co-investigators [12]; the microgranular storage pool deficiency has a normal distribution of dense granule development in contrast to the empty sac/granule syndrome.

The uranaffin reaction used for this study was a modified technique (slightly lower pH and decreased reaction time). The portions of glutaraldehyde-fixed buffy coat were immersed in saturated aqueous uranyl acetate (pH 3.3) for two hours, followed by a graded series of ethanolic dehydration steps, a transitional dehydration step with absolute acetone and the subsequent infiltration and embedment in Spurr’s epoxy resin [33,34]. The tissues prepared in this manner were sectioned for both routine TEM and for HVEM. The platelets subjected to the uranaffin reaction were evaluated by routine TEM and classified based upon their morphology as described by Weiss et al. [12]. The granules with electron-dense material occupying more than 50% of their interiors were classified as Type 1 (Figure 2A); solid granules occupying less than 50% of the membrane-limited vacuoles were classified as Type 2 (Figure 2A); granules with fragmented cores were classified as Type 3 (Figure 2A); and “empty sacs” vacuoles with uranaffin-positive membranes without an electron-dense interior were classified as Type 4 (Figure 2A) [12].

### 2.6. High-Voltage Transmission Electron Microscopy (HVEM)

**Figure 3:**
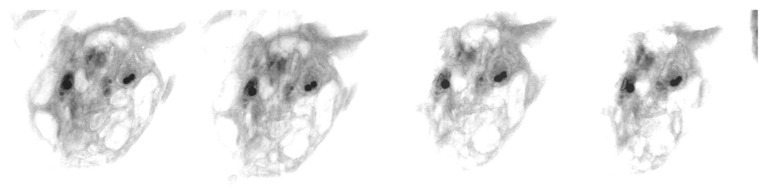
Thick sections of a platelet observed in a tilt series using a high voltage TEM. This demonstrates the spherical nature of dense granules; overlapping dense granules (DGs) may appear oblong.

Buffy coat samples processed for the uranaffin reaction from 10 normal subjects and 10 δ-SPD cases and embedded in epoxy were sectioned 0.75–1.0 micron thick for HVEM. The “thick” sections were placed on copper mesh electron microscopy grids and air dried without any post-section staining procedures. These 20 samples were evaluated by HVEM (1.0MV) at the NIH Biotechnology Resource at the Wadsworth Center in Albany, New York, to determine platelet dense granule shape and size from a series of electron photomicrographs recorded at different specimen tilt angles (Figure 3). The investigation included the image analysis of corresponding wmTEM photographs for comparison with the HVEM images performed as a blind study.

### 2.7. Total Adenosine Nucleotide Content

The extraction and determination of platelet adenosine nucleotide content were performed by standard methods [3,35,36,37,38]. A 500 µL sample of PRP was mixed with 450 µL of 96% ethanol (EtOH) and 50 µL of 0.1M EDTA, pH 7.4, for 10 s, and centrifuged for 15 min at 14,000× *g*. The supernatant was stored at −25 °C until assayed.

The ATP and ADP contents of the platelets were determined by the bioluminescence of firefly luciferase method. Using a Lumat LB9501-2 Luminometer (Wallac Inc., Gaithersburg, MD, USA) and known quantities of ATP (Sigma ATP Bioluminescent Assay Kit (Cat. FL-AA), Sigma Chemical Co., St. Louis, MO, USA), a standard curve was determined prior to the analysis of adenine nucleotides extracted from PRP preparations. The procedure determined the total ATP and ADP concentrations extracted from the lysed platelets and was not used to determine the adenine nucleotides contained solely within the storage pool dense granules (DGs). The ATP analysis was quantified first. To determine the ADP content of the platelets, the extracted ADP was converted to ATP using creatinine phosphokinase and phosphocreatine, and quantified using the bioluminescence assay [3]. The amount of ADP in the lysate could then be determined by the subtraction of the original ATP concentration in the lysates from the results of the second assay that included ADP converted to ATP [3].

### 2.8. Image Analysis

For this study, a total of 100 contiguous air-dried platelets were assessed for DG content; platelets (PL) that were partially obscured by a grid bar or that exhibited preparation artifacts were excluded from the evaluation. The total number of DGs enumerated within the whole mounted platelets included in the assessment were divided by 100 (total PL observed) and the average number of DG/PL was determined. During the enumeration of DGs, 12 random platelet fields were photographed at 10,200X using a Philips 410LS TEM. The platelets were representative of the preparation and included 12–20 platelets for subsequent image analysis to accurately measure the DG diameter and to calculate the DG volume. The platelets that were photographed had at least 4 DG/PL for purposes of generating a reasonable *N* for statistical validity. No statistical difference was found for DG size between DGs measured in platelets with 1–4 DG/PL versus platelets having more than 10 DG/PL. The image analysis employed NIH Image 1.57^®^, Image Pro 5.1^©^ (MediaCybernetics, Silver Spring, MD, USA) and proprietary software written by two of the co-authors (BR and SR). The diameter of each digitized DG was used to calculate the volume of each DG (4/3 πr^3^). The mean DG volume (MDGV) for each platelet was calculated from the sum of DG values divided by the number of DGs evaluated. The total dense granule volume (TDGV) was calculated by multiplying the MDGV by the mean number of DG per PL. The confirmation of DG spherical morphology used 1 µm thick plastic sections of uranaffin-reacted PLs photographed at different specimen tilt angles with the HVEM (Figure 3). Using Image Pro 10^©^, another co-author (MR) utilized artificial intelligent macros to validate all the measurements obtained initially for the 13 index cases (Supplementary Figure S1: Comparing AI vs. Manual Measurements of Index Cases, Supplementary Table S1: AI, Supplementary Table S2: Total Group DG Numbers and Volumes).

### 2.9. Statistical Analysis

The values for the average DG/PL and TDGV/PL were calculated. The values for the “normal” (control) subjects were obtained and averaged for comparison with the values calculated for individuals who had a bleeding history. For each calculation, the normal values were based upon a range including 3 standard errors of the mean (SE) from the average of all control samples. Student *t* and chi square tests were performed using SigmaPlot^©^ 12 (SSPS, Inc., Chicago, IL, USA).

## 3. Results

The platelets of 49 normal (control) subjects contained 4.64 ± 0.11 (SE) DG/PL, similar to the values previously reported [4,12,13,38,39,40]. The mean diameter of the control dense granules was 143.14 ± 1.56 nm (Table 2) which was also similar to that previously reported [32,41].

This study included 1080 individuals, none of whom were thrombocytopenic and had platelet electron microscopy (EM) and image analysis performed for the evaluation of unexplained bleeding. Four hundred and ninety-nine (499) patients had a classic dense granule deficiency with an average number of DG/PL of 2.25 ± 0.04 (Table 2). The average diameter of their dense granules (151.91 ± 23.74 nm) was similar to the average diameter of normal subjects’ DGs (143.0 ± 1.56 nm (SE)). Three hundred and twenty-eight (328) patients having bleeding symptoms were all found to be within normal limits using the analyses employed in this study. A total of 88 individuals were found to have an elevated number of DGs (6.64 ± 0.11 DG/PL) with a normal diameter (141.76 ± 0.17 nm) but significantly elevated TDGV (14.23 ± 0.54 × 10^6^ nm^3^) compared to all the other groups which is of unknown significance; three of these individuals were known to be hyper-aggregable, but most presented with bleeding symptoms. One hundred and sixty-five (165) patients were found to have an average dense granule diameter more than three standard errors of the mean below the mean of normal subjects. Their platelets contained 4.31 ± 0.36 DG/PL (no statistical difference) with a mean dense granule diameter of 123.36 ± 0.86 nm (*p* < 0.001) compared to the controls. The calculated mean volume of their DGs was significantly decreased compared to normal (*p* < 0.001). The total DG storage pool volume per PL was 5.99 ± 1.13 x 10^6^ nm^3^, which was significantly decreased compared to the value obtained for the normal subjects (10.79 ± 0.39 × 10^6^ nm^3^, *p* < 0.001). The TDGV per PL for these individuals was found to be similar in magnitude to that seen in classic platelet dense granule deficiency (3.88 ± 1.06 × 10^6^ nm^3^). We have termed this defect δ-MGSPD, a dense granule storage pool volume deficiency (SPVD). There was no correlation between the platelet count, the DG number, nor the total volume of DG/PL for individuals with SPVD. For both normal subjects and patients with classic dense granule deficiency (DGD), there was a very strong relationship (r = 0.87) between the dense granule number and the total volume of dense granules (Table 2). Using the dense granule number and the total volume of dense granules, individuals with δ-MGSPD were easily separable from the continuum of normal subjects and patients with DGD.

**Figure 4:**
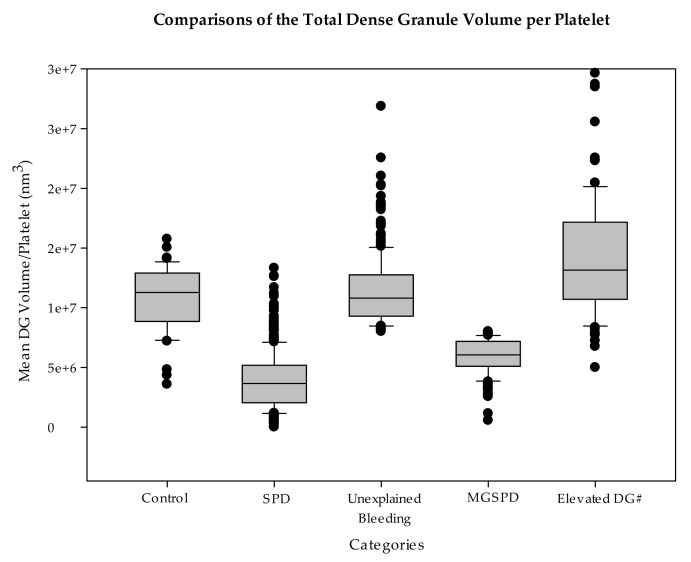
The graph illustrates that the patients categorized as microgranular storage pool deficient (δ-MGSPD) are similar in total dense granule volume (TDGV) per platelet to classic dense granule deficiency (δ-SPD) when compared to the TDGV of control subjects, unexplained bleeding patients and those who were found to have elevated numbers of granules. This graph includes all the subjects included in the study.

The distribution of the dense granule size was also evaluated to determine if the decrease in the dense granule volume in δ-MGSPD was due to a loss of the larger sized granules or whether the volume decrease was due to all the granules being smaller than normal. Patients with δ-MGSPD were found to have a population of platelet dense granules that were smaller than those of the control group. Although the volume of the largest dense granules in individuals with δ-MGSPD was similar to normal subjects, the percentage of larger granules was decreased. A scatter box plot of patients and control subjects is shown in Figure 4 to exemplify these differences; individuals with δ-MGSPD had a dense granule distribution that shifted to smaller sizes compared to normal subjects. Individuals with δ-MGSPD had 59.4% of their granules <1.5 × 10^6^ nm^3^ in volume, whereas none of the granules of normal subjects were as small. Forty-three percent (43%) of the “classic” SPD patients had a dense granule volume (DGV) <1.5 × 10^6^ nm^3^.

In addition to our morphometric calculation of a total storage pool volume, we classified DGs using the uranaffin reaction described by Richards, et al. [33] and later employed by Weiss et al. (1993) to determine the distribution of the DG type [12]. Figure 2 shows that individuals classified as δ-MGSPD have a granule “type” distribution similar to that of normal individuals and this classification is not similar to that of empty granule/empty sac syndrome [12,40].

Since the calculation of platelet dense granule volume was based on the presumption that platelet dense granules are spherical, we calculated their volume by standard formulas of solid geometry. To confirm the assumption, we examined the three-dimensional shape of DGs using HVEM tilt series of uranaffin-eacted epoxy sections (Figure 4). All the uranaffin-enhanced DGs were found to be spherical (excluding an occasional granule undergoing degranulation). Although some granules initially appeared to be rod-shaped or elliptical in morphology, the subsequent tilting of the sample section relative to the beam demonstrated that these granules were actually two or more granules that were superimposed with similar positions in the specimen plane, but were at different locations within the thickness of the sample. When the specimen was tilted to different orientations relative to the beam, two distinct particles with circular cross-sections were revealed. These data confirmed our hypothesis that platelet dense granules are indeed spherical and validated our volume calculations.

Given the clinical similarity between the individuals with DGD and δ-MGSPD, and given the similarity in total DGV between these two disorders, we next determined the ADP and ATP concentrations in the platelets of individuals with δ-MGSPD. If δ-MGSPD is a bleeding disorder analogous to DGD, then the ADP and ATP contents of these platelets would be expected to be decreased. In the normal subjects, there was a very strong correlation between the ADP content and the TDGV (r = 0.91), suggesting that the ADP assay was primarily reflecting the ADP content of the dense granules (Table 2). The ADP and ATP contents of platelets from individuals with δ-MGSPD were decreased compared to the normal platelets. The ADP and ATP concentration in δ-MGSPD were similar to those seen in classic DGD (Table 2). For the normal subjects and individuals with δ-MGSPD, there was a strong correlation between the TDGV and the concentrations of ADP (r = 0.56), ATP and the ADP + ATP content (r = 0.70). Individuals with δ-MGSPD had ADP and ADP + ATP concentrations that were indistinguishable from those of individuals with dense granule deficiency and the two groups had similar TDGVs (Table 2).

## 4. Discussion

At the onset of our studies of patients having unexplained bleeding, we used the platelet whole mount technique for electron microscopy to determine whether these patients had a normal number of DG. We sought to establish that the normal subjects assayed in our laboratory had similar values to control subjects cited in the literature [13,23,24,25,29]. There are a number of assays that have been used to evaluate unexplained bleeding including lumiaggregometry, the measurement of 5-HT content, and mepacrine assays as other means for the diagnosis of δ-SPD [41,42,43,44,45,46,47]; however, the platelet whole mount TEM techniques remains the gold standard [41,48]. The term “gold standard” is used in other contexts as well, such as for the diagnosis of inherited platelet secretion disorders, whereas radioactive serotonin incorporation and release is cited as the gold standard for δ-granule release [49]. Since the technique was first described by Bull and refined by White, the technique has been reliably utilized for 50 years for objective morphologic, not functional, assessments [13,23,24,26]. The number of DG/PL that we have established as normal in our laboratory is 4.54 ± 0.29 and that the lower limit for a normal DG number, at three standard errors from the mean, is 3.68 DG/PL; these values are similar to those published in other studies of δ-SPD [13,23,24,26]. These findings are at odds with a single report suggesting that the normal range of DGs is significantly lower at 1.2-4.0 DG/PL, which is radically different than what the rest of the literature suggests. Those investigators utilized energy dispersive X-ray (EDX) analysis to categorize DGs based upon the identification of calcium and phosphorus, both elements that are known to concentrate in DGs [50]. The use of EDX for the determination of SPD is novel and an infrequently available technique in clinical electron microscopy (EM) laboratories.

For those individuals found to have a normal to elevated number of DGs, we were able to determine that 165/581 (28.4%) had a mean DG diameter statistically smaller than that of our normal subject group. The adenine nucleotide pool was found to be very similar to the means of δ-SPD patients and significantly less than the control values (Table 2).

Individuals having a bleeding tendency in association with a normal platelet count should be evaluated for von Willebrand disease. If normal, a qualitative platelet defect or δ-SPD should be considered. δ-SPD has historically been diagnosed using platelet aggregation assays, by the firefly luciferase adenine nucleotide assay, whole mount electron microscopy and more recently by flow cytometry using quinacrine (mepacrine)-labeled platelets [10,51,52]. We report the use of image analysis to assess the dense granule volume in patients with a significant bleeding history who appear to have a normal number of dense granules.

Platelet dense granule or delta granule (DG) deficiency (δ-SPD) is a heterogeneous bleeding diathesis, often characterized by prolonged bleeding time (BT) or closure time, variable abnormalities in platelet aggregation, decreased adenine nucleotides and decreased DGs by EM [31,32,50,51,52,53,54,55,56,57]. In our experience, it is not infrequent to find that patients with a significant bleeding history may not be diagnosed with any particular disorder following platelet aggregation and release studies. Such patients are often labeled as being “VWD-like” and remain a diagnostic enigma presently labeled as “Bleeding of Unknown Cause” [58,59,60,61]. Further research is needed to determine whether the prevalence of δ-SPD and its microgranular variant (δ-MGSPD) is an even more common entity than VWD as a cause of unexplained mucocutaneous bleeding. The advent of whole exome sequencing should also better define such patients [62].

The limitations of our study include a number of assessments that were not made such as age and sex correlation, ethnic background and family studies, the use of a bleeding assessment tool for the objective scoring of bleeding symptom severity, and the review of each patient’s complete blood cell count and prior hemostasis testing. Many of the patients included in this study had samples that had been referred from a number of other hospitals and health centers without accompanying medical history. Blood was also submitted in acid citrate dextrose (ACD) tubes, diluting some of the values obtained by CBC. As previously stated, we have reported that blood samples in ACD tubes are stable for at least 48 h post-venipuncture for the assessment of DGs and/or VWD profile testing [30]. The adenine nucleotide results included in this report, while limited, were critically scrutinized for accuracy with adequate quality control.

The essential point to be concluded from this study is that δ-SPD may be a significant etiology for patients presenting with bleeding histories that are difficult to diagnose by traditional means. In our experience, it is apparent that δ-SPD can clinically mimic von Willebrand disease, as both disorders present with the same symptoms. Many of the patients that we have evaluated have previously been “labeled” as having a “von Willebrand-like syndrome” as they have a normal aggregation assays, normal von Willebrand antigens and activity, collagen-binding assays and factor VIII clotting factor activity. Previous studies have reported the disorder that cannot be excluded with simple aggregation tests [23,24,25]. There have been no population-based studies to determine the incidence of δ-SPD and the prevalence of inherited platelet disorders is unknown [63]. Unfortunately, a specific etiology of the disorder has yet to be established. The inheritance pattern is often described as autosomal dominant, but both autosomal recessive and acquired SPDs have been reported. Delta granule storage pool deficiency is well documented in Hermansky–Pudlak, Chediak–Higashi, and Wiskott–Aldrich syndromes, all of which have known mutations and none of which are common to one another. The etiology of δ-SPD appears to be multifactorial and it is often unrecognized, especially when extensive laboratory workup, including whole mount EM, fails to identify the cause of unexplained bleeding. The utilization of TEM cannot determine functional secretions defects and our group of patients with normal numbers of DGs and TDGV may in fact have a functional defect. The results of the present study demonstrate that variations of δ-SPD do exist. Weiss et al. [12] described a variant δ-SPD in which DGs were conspicuously absent but had numerous “empty” uranaffin reactive membrane limited vacuoles which was subsequently termed the Empty Sac Syndrome by McNicol et al. in 1994 [40]. We now report a new variant δ-SPD which we have termed “microgranular delta storage pool disease” (δ-MGSPD) in which prominent bleeding history is evident, aggregation studies may/may not be abnormal, and the wmTEM of the dense granule number is within normal limits, but the mean diameter of DGs is significantly less than that of normal subjects. Further scrutiny of the platelet DGs including image analysis for DG measurement and the determination of the adenine nucleotide content may be required to establish an etiology for some cases of undiagnosed bleeding disorders. It may even go undiagnosed following routine electron microscopic evaluation. These findings may also be important as a basis to investigate possible altered mechanism(s) of platelet formation/maturation and/or function that lead to the qualitative platelet dysfunction related to diminished dense granule constituents.

## Figures and Tables

**Table 1:** Demographics of the index patient cohort.

**A. Clinical Features of Individuals with Platelet Microgranular Storage Pool Deficiency**				
**Subject**	**Age**	**Sex**	**Family History of Bleeding**	**Patient Bleeding History**
1	32	F	Yes	Postpartum, cervical biopsy, menorrhagia
2	13	F	Yes	Bruising, menometrorrhagia
3	54	M	Yes	Tonsillectomy, dental extraction, vasectomy
4	34	F	Yes	Bruising, epistaxis, menorrhagia, perimenstrual bruising
5	43	F	Yes	Postpartum ×2, c-section
6	48	M	Yes	Laminectomy
7	80	M	No	CABG/aortic valve replacement
8	56	F	Yes	Bruising (exacerbated by ASA), epistaxis
9	50	F	No	Bruising
10	53	F	No	Bruising (exacerbated by ASA, NSAID), epistaxis, dental extraction
11	60	F	Yes	Bruising, epistaxis, menorrhagia
12	46	F	Yes	Bruising, epistaxis, menorrhagia, postpartum, dental extraction, breast biopsy, hemicolectomy, arthroscopy
13	76	M	No	Tonsillectomy, colonic polypectomy ×2
**B. Laboratory Features in Index Patients with Platelet Microgranular Storage Pool Deficiency**				
**Subject**	**Bleeding Time (Normal <8.5 min)**		**ADP**	**Platelet Aggregation Results** **Epinephrine**
1	9, 12		Decreased	Absent
2	6		Normal	Normal
3			Normal	Normal
4	5		Normal	Normal
5	>20		Decreased	Normal
6	6		Decreased	Decreased
7	12		Clotted	Clotted
8	>20		Normal	Decreased
9			Decreased	Decreased
10	>20		Decreased	Decreased
11	>26		Normal	Decreased
12			Decreased	Decreased
13	6		Normal	Normal

**Table 2:** Characterization of the studied subjects.

	Control Subjects	Dense Granule Deficiency (δ-SPD)	Microgranular Storage Pool Deficiency (δ-MGSPD)		Unexplained Bleeding with Normal Values	Unexplained Bleeding with Elevated DGs
Patients with Bleeding History (*n* = 1080)	*n* = 49	*n* = 499	*n* = 165	*n* = 328		*n* = 88
Granule Characterization						
Dense Granules/Platelet	4.64 ± 0.11	2.25 ± 0.04 *	4.31±0.36	4.55 ± 0.03		6.64 ± 0.11 *
Dense Granule Diameter (nm)	143.14± 1.56	151.9 ± 2.37	123.4 ± 0.86 *	150.0 ± 0.68		141.76 ± 0.17
Volume/Granule (10^6^ nm^3^)	2.32 ± 0.71	2.68 ± 0.29 **	1.40 ± 0.26 *	2.55 ± 0.39		2.15 ± 0.76
Total DG Volume/PL (10^6^ nm^3^)	10.79 ± 0.42	3.88 ± 1.06 *	5.99 ± 1.13 *	11.59 ± 0.21		14.23 ± 0.54 **
Adenine Nucleotides	(*n* = 42)	(*n* = 6)	(*n* = 8)	(*n* = 18)		ND
ATP (µM/10^11^ platelets)	4.18 ± 0.20	3.68 ± 0.63	3.26 ± 1.69	4.72 ± 0.89		
ADP (µM/10^11^ platelets)	2.38 ± 0.14	1.52 ± 0.49	1.65 ± 0.68	2.92 ± 0.34		
ATP/ADP	1.83	2.42	2.05	1.71		

Mann–Whitney Rank Sum: * p < 0.001, value is significantly different than the control value ** *p* < 0.05, value is increased compared to the control value.

## Supplementary Materials

The following are available online at https://www.mdpi.com/2077-0383/9/6/1734/s1, Supplementary Figure S1: Comparing AI vs. Manual Measurements of Index Cases, Supplementary Table S1: AI, Supplementary Table S2: Total Group DG Numbers and Volumes.
